# Reply From Authors: How a cell dies matters and how to evaluate it also matters

**DOI:** 10.1016/j.xjon.2022.05.016

**Published:** 2022-06-02

**Authors:** Satoshi Ueda, Toyofumi F. Chen-Yoshikawa, Satona Tanaka, Yoshito Yamada, Daisuke Nakajima, Akihiro Ohsumi, Hiroshi Date

**Affiliations:** a Department of Thoracic Surgery, Kyoto University Graduate School of Medicine, Kyoto, Japan; b Department of Thoracic Surgery, Nagoya University Graduate School of Medicine, Nagoya, Japan

Reply to the Editor:

**Figure:**
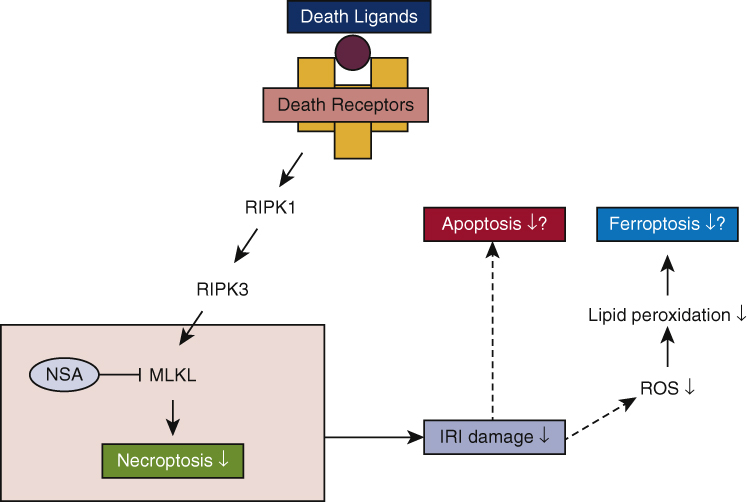

The authors reported no conflicts of interest.

The *Journal* policy requires editors and reviewers to disclose conflicts of interest and to decline handling or reviewing manuscripts for which they may have a conflict of interest. The editors and reviewers of this article have no conflicts of interest.

This study aimed to investigate the effect of necrosulfonamide (NSA), a mixed-lineage kinase domain-like protein (MLKL) inhibitor, on pulmonary ischemia-reperfusion injury (IRI) in a simple and validated in vivo model. NSA treatment showed a protective effect on pulmonary IRI, because of improvement of physiological parameters and histological findings and a lower level of proinflammatory cytokines were observed in the NSA-treated group.^1^

We adopted double-labeling with cleaved caspase-3 and terminal deoxynucleotidyl transferase- dUTP nick-end labeling (TUNEL) to evaluate necroptotic/apoptotic cells, as previously described by others.^2^ TUNEL-positive and caspase–3-negative cells were defined as necroptotic cells.^2^ As Lin and colleagues reported in a “Letter to the Editor,”^3^ this method might count other cell death that is caspase-independent such as ferroptosis. Ferroptosis is a recently established mode of programmed cell death that is dependent on iron and characterized by the accumulation of lipid peroxides. A decrease in TUNEL-positive and caspase–3-negative cells might indicate a decrease in ferroptosis as well as necroptosis, however, to the best of our knowledge, there are no specific markers that are applicable in rat lung tissue to distinguish ferroptosis from other types of cell death. In the past, in experimental reports describing IRI,^4^, ^5^ cell death inhibited by necrostatin-1 and ferrostatin-1 was defined as necroptosis and ferroptosis, respectively. Necrostatin-1 inhibits receptor-interacting protein kinase-1 (RIPK1) and NSA inhibits MLKL, which is the downstream of RIPK1 and regulates necroptosis. We concluded that the protective effect of NSA on pulmonary IRI was mainly caused by the inhibition of MLKL-mediated cell death, namely, necroptosis. A decrease in other types of cell death such as ferroptosis and apoptosis might be observed as a result of attenuated lung damage in the NSA-treated group.

How a cell dies matters and how to evaluate it also matters. In the future, it is necessary to find a way to more rigorously distinguish necroptosis from ferroptosis and other cell death to advance research on programmed cell death that is applicable in various settings of experimental models.
